# Frontal Plane Motion of the Pelvis and Hip during Gait Stance Discriminates Children with Diplegia Levels I and II of the GMFCS

**DOI:** 10.5402/2012/163039

**Published:** 2012-06-25

**Authors:** Renata Noce Kirkwood, Rosa de Lourdes Lima Dias Franco, Sheyla Cavalcanti Furtado, Ana Maria Forti Barela, Kevin John Deluzio, Marisa Cotta Mancini

**Affiliations:** ^1^Department of Physical Therapy, Universidade Federal de Minas Gerais, 31270-901 Belo Horizonte, MG, Brazil; ^2^Faculty of Engineering and Applied Science, Mechanical and Materials Engineering, Queen's University, Kingston, ON, Canada K7L 3N6; ^3^Biologic Science and Health Institute, Pontifícia Universidade Católica de Minas Gerais, 30535-901 Belo Horizonte, MG, Brazil; ^4^Institute of Physical Activity and Sports Science, Universidade Cruzeiro do Sul, 01506-000 São Paulo, SP, Brazil; ^5^Department of Occupational Therapy, Universidade Federal de Minas Gerais, 31270-901 Belo Horizonte, MG, Brazil

## Abstract

*Objective*. To determine if gait waveform could discriminate children with diplegic cerebral palsy of the GMFCS levels I and II. *Patients*. Twenty-two children with diplegia, 11 classified as level I and 11 as level II of the GMFCS, aged 7 to 12 years. *Methods*. Gait kinematics included angular displacement of the pelvis and lower limb joints during the stance phase. Principal components (PCs) analyses followed by discriminant analysis were conducted. *Results*. PC1s of the pelvis and hip in the frontal plane differ significantly between groups and captured 80.5% and 86.1% of the variance, respectively. PC1s captured the magnitude of the pelvic obliquity and hip adduction angle during the stance phase. Children GMFCS level II walked with reduced pelvic obliquity and hip adduction angles, and these variables could discriminate the groups with a cross-validation of 95.5%. *Conclusion*. Reduced pelvic obliquity and hip adduction were observed between children GMFCS level II compared to level I. These results could help the classification process of mild-to-moderate children with diplegia. In addition, it highlights the importance of rehabilitation programs designed to improve pelvic and hip mobility in the frontal plane of diplegic cerebral palsy children level II of the GMFCS.

## 1. Introduction

Cerebral palsy is a nonprogressive central nervous system disorder that results in physical impairments and functional limitations that change as the children grow older [1]. Among a large number of instruments [2–4], for measuring the physical ability of children with CP, the *Gross Motor Function Classification System *(GMFCS) introduced by Palisano et al. in 1997 [5] has been widely applied in clinical and research settings [6]. The GMFCS is a five-level classification system that identifies abilities and functional limitations, based on the need of assistive devices of the cerebral palsy child, during self-initiated movements, such as walking and sitting [5]. The system application is quick and easy and it gives a brief description of which level the child resembles based on his/her current gross motor function.

The reliability and validity of the GMFCS in differentiating cerebral palsy children with different functional levels have been reported [1]. Similarly, the stability of the system over time proved to be very consistent, suggesting that the GMFCS could be used routinely in clinical practice to follow children with cerebral palsy [7]. However, due to the heterogeneous nature of cerebral palsy, some overlap between levels I and II has been observed and, indeed, anticipated by the authors [2, 5, 7]. GMFCS level I is associated with children with persistent neuromotor abnormalities not as severe as children from level II. Overlap between levels occurred more often when deciding if a child has limitations walking outdoors, going up stairs, jumping, or running [5]. Many studies aimed to determine which outcome tools could assist clinicians and researchers to improve the classification levels I and II of the GMFCS [1, 4, 8]. Correct classification at clinical settings has been corroborated by tests such as the GMFM-66, gait velocity, and the WeeFIM Mobility [8, 9].

Child with cerebral palsy often develops changes in muscle length over time, more common at the hip and knee flexors and at the ankle dorsiflexors [10, 11]. Kilgour and colleagues (2005) using passive range of motion tests reported that diplegic children levels I and II of the GMFCS had minimal loss of hip extension compared to a matched control group [12]. Since the GMFCS classification is based on functional activities, it would be more interesting to obtain measurements during dynamic activities. In addition, it is expected that muscle shortness is more advanced in diplegic children level II than level I of the GMFCS. Therefore, range of motion comparison between levels I and II is also relevant. These muscles changes might affect the range and amplitude of the lower limb during dynamic activities such as gait. However, to date, none of the studies tried to discriminate GMFCS levels I and II according to the angular displacement of the pelvis, hip, knee, and ankle/foot complex during gait. Instrumented gait analysis is a complex procedure and a highly costly diagnostic tool; however, the kinematic data obtained provides quantitative information on gait abnormalities that cannot be detected during visual observation. Raising the knowledge of the gait biomechanical differences between child with cerebral palsy level I and II of the GMFCS can improve observational gait analysis skills and, at the same time, improve classification accuracy and more coherent physical therapy approaches based on the functional status of children with cerebral palsy.

Therefore, the research questions for this study were: (1) Are there differences in the kinematics gait profiles of the pelvis and lower limb joints during gait between diplegic cerebral palsy children classified as GMFCS levels I and II? (2) If differences in the kinematics were found, which ones would be the most discriminatory between these groups?

## 2. Method

A cross-sectional observational study was conducted with diplegic cerebral palsy children classified by a trained physical therapist, as GMFCS levels I and II. The intra-rater reliability of the physical therapist in assessing the GMFCS levels was excellent (ICC = 0.941). All children were community ambulates from outpatient clinics and, together with their parents or guardians, were invited to participate in the study. The temporal and spatial gait parameters and kinematics of the pelvis, hip, knee, and ankle/foot joints were collected on one day in a laboratory. The present study received approval from the Ethics Committee of the Universidade Federal de Minas Gerais, process number ETIC 088/04. Prior to participation, all procedures were explained to the child and his/her parent or guardian and an informed written consent was obtained.

### 2.1. Participants

Twenty-two (22) diplegic cerebral palsy children were included in the study, 15 male and 7 females between the ages of 7 and 12, who could ambulate independently without assistive devices for a minimum of 6 meters without resting. Patients were excluded if they had other neurological diseases, botulin injections, or history of orthopedic surgery in the past six months. Characteristics of the participants, such as age (years), height (m), and body mass index (BMI, Kg/m^2^), were obtained in order to describe the anthropometrics of the groups.

### 2.2. Outcome Measures

Three-dimensional kinematics of the right lower limb (hip, knee, and ankle/foot joints) and pelvis during the stance phase of the gait cycle were obtained with a six camera motion analysis system (*Motion Capture Unit-QUALISYS MEDICAL AB *411 12, *Gothenburg, Sweden*). The children walked barefoot over a 6-meter walkway, for an average of 9 (SD = 3.1) trials at their natural speed. The trials were collected continuously since none of the children asked for rest. The average number of trials from each child was used in the analysis. Reflective markers and clusters of tracking markers were used to determine coordinate systems and motions of the pelvis, thigh, shank, and ankle/foot segments according to recommendations for minimizing soft tissue artifacts [13]. A footswitch synchronized to the motion system was fixed under the children's foot to determine contact to and loss of contact from the walking surface, and consequently, delimiting stance and swing phases during gait cycle.

### 2.3. Data Reduction

The resulting data were processed through the Visual 3D Motion Analysis Software (C-Motion, Inc, Rockville, Maryland) where the rigid segments corresponding to the pelvis, shank, thigh, and ankle/foot segments were first created. The position of the reflective anatomical markers were used for attributing coordinate systems for each segment and were located at left and right iliac crest, left and right greater trochanter, medial and lateral epicondyle of the femur, medial and lateral malleoli, 1st and 5th head of the metatarsus, and calcaneal tuberosity. One rigid cluster with 4 noncollinear markers was placed at the base of the sacrum and two nonrigid clusters with 3 noncollinear markers were placed at the medial side of the thigh and shank. Data were smoothed using a zero-lag fourth-order Butterworth low pass filter with a cut-off frequency of 6 Hz [14].

Three-dimensional angular motion was calculated using the Cardan sequence, defined as the orientation coordinate system of one segment in relation to the orientation of the coordinate system of the adjacent segment. The pelvic angle was computed using the global coordinates of the laboratory as reference. The hip, knee, and ankle/foot joint angles were obtained using as reference the pelvis, thigh, and the shank segments, respectively. The sign convention used in defining the clinical rotation angles were as follows: (1) flexion of the hip and knee, anterior tilt of the pelvis, and ankle dorsiflexion, all of which occur about the lateral-medial or *X*-axis and were positive angles; (2) adduction of the hip and knee, pelvic obliquity (meaning the height of the iliac crest of the stance foot higher in relation to the height of the iliac crest of the opposite foot) and internal rotation of the ankle/foot complex, all of which occur about the anterior-posterior or *Y*-axis and were considered positive angles; (3) internal rotation of the pelvis, hip and knee joints and adduction of the ankle/foot complex occurs about the distal-proximal or *Z*-axis and all were positive angles. Gait velocity (m/s), stride length (m), and cycle time (s) for the entire gait cycle, and swing and stance time (s), were also obtained.

### 2.4. Statistical Analysis

Baseline characteristics of participants are presented as values, means, and standard deviations (SD). The mean difference between the groups with a 95% CI of the subject's characteristics and temporal and spatial gait parameters were also obtained. A principal component analysis (PCA) was applied to the stance phase of the gait waveforms to reduce the data and explore the profiles characterizing typical functions between levels I and II of the GMFCS [15, 16]. In PCA, multidimensionality of highly complex data is reduced with minimal loss of information [17]. This technique determines a linear combination of the original variables that are used to summarize a new set of variables called principal components, which are uncorrelated and ordered so that the first component retains most of the variation present in the original data [18]. Therefore, each principal component represents a specific feature of the waveform data [17].

The criteria to choose the number of principal components was 90% of the total sample variance [19]. For each PC extracted, a score is calculated for each subject that aids in describing the meaning of the variation component according to the characteristics of each group. The higher the score, the more correlated the subject's waveform is with a specific PC [19]. To interpret the components, two waveforms were created based on the mean waveform ± one standard deviation of the PC scores times the loading vector for each PC [20].

The principal component scores were analyzed using Student's *t*-test with Bonferroni correction for difference between groups. Following, a stepwise discriminant procedure was used to the significant PC scores. The PCs retained after the discriminant analysis were described according to the discriminant function coefficient to determine its relative importance in separating the groups [21]. All analyzes considered a significance level of 0.05.

## 3. Results

Group GMFCS level I had 11 children with an average age of 9.1 years (SD: 2.3), average height of 1.3 m (SD: 0.1) and BMI of 16.7 kg/m^2^ (SD: 0.2). Group level II, also with 11 children, had average age of 9.8 years (SD: 2.1), height of 1.3 m (SD: 0.1), and BMI of 17.2 kg/m^2^ (SD: 3.8, Table 1). Table 2 describes the temporal and spatial parameters between groups with the 95% confidence interval showing no significant difference between groups.

Principal component analyses were carried out for three-dimensional angular displacement of the pelvis, hip, knee, and ankle/foot complex. The PC scores generated for each subject in each component were tested for differences between groups. The results showed that only the scores from the first component (PC1) of the pelvis and hip joint in the frontal plane were statistically different between the groups (Table 3, *P* < .05). No difference was found in the curve profiles of the knee joint and ankle/foot complex.

**Figure 1:**
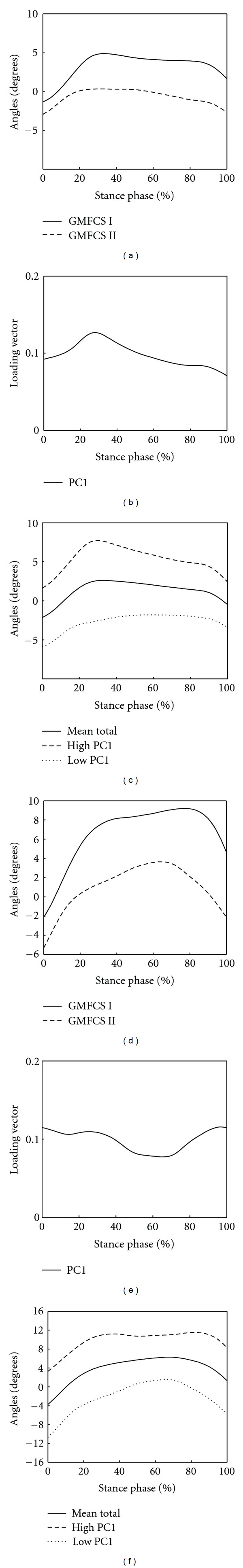
(a) Average angular displacement of pelvic obliquity during the stance phase of gait of the GMFCS groups' level I and II (b) loading vector or PC1 of pelvic obliquity; (c) mean waveforms of pelvic obliquity and high and low scores represented by adding or subtracting 1SD of the scores times the correspondent loading vector; (d) average angular displacement of the hip adduction during the stance phase of gait from the GMFCS levels I e II; (e) loading vector from hip adduction/abduction angle in the frontal plane; (f) mean waveforms of hip adduction/abduction angle and high and low scores represented by adding or subtracting 1SD of the scores times the correspondent loading vector.

At the pelvis, two components explained 94.6% of the variability of the data. Figure 1(a) shows the angular displacement of the pelvis during the stance phase (normalized to 100%) between level I and II groups of the GMFCS. The PC1 explained 80.5% and the PC2 14.1% of the variance. The coefficient of PC1 has all positive values (Figure 1(b)); therefore, it captures the magnitude of the pelvic obliquity angle in the frontal plane during the stance. Figure 1(c) shows the mean waveform and the high and low curves created based on the mean waveform ± one standard deviation of the PC1 score times the loading vector for each PC1. The results confirm that, on average, diplegic PC children level II, during the stance phase of the gait, walked with reduced pelvic obliquity in comparison with children from group level I. The average range of pelvic obliquity during the stance phase of the gait cycle for group level I was 6.2° and for level II was 3.3°.

Figure 1(d) shows the average angular displacement of the hip joint in the frontal plane between groups during the stance phase of the gait cycle. At the hip joint, two PCs were extracted with PC1 explaining 86.1% and PC2 8.5%, a total of 94.6% of variance explained. PC1, with all positive values, measured the magnitude of the adduction angle of the hip (Figure 1(e)). The mean waveform and the high and low waveforms are shown on Figure 1(f) and confirm that children in GMFCS level II presented reduced hip adduction during the stance phase when compared to cerebral palsy children in level I. The average range of hip adduction/abduction during the stance phase of the gait cycle for group level I was 11.3° and for level II was 8.9°.

A stepwise discriminant analysis was conducted with the PC1s of the pelvis and hip in the frontal plane. Wilk's Lambda score was significant (⋀ = .217, *χ* ^2^ (2, *N* = 22) = 29.002, *P* = .000) and showed that both pelvic obliquity and hip abduction angle are stronger discriminant variables, with 95.5% cross-validation. The magnitude of the coefficients of the PCs in the standardized canonical discriminant function showed that pelvic obliquity has higher impact on separating the groups (0.711) followed by hip abduction angle (−0.654) during the stance phase of gait. 

## 4. Discussion

To our knowledge, this is the first study that compared the angular displacement of the pelvis and lower limb joints, between diplegic cerebral palsy children classified according to the GMFCS as levels I and II. The results demonstrated that children with diplegia level II of the GMFCS significantly reduce the amplitude of pelvic obliquity and hip adduction angles during the stance phase of the gait cycle. Gait velocity, stride length, stance/swing and cycle time were similar in outcome for both groups.

Children classified as GMFCS Level I of ages between 7 and 12 years, are expected to walk independently inside and outside their homes, to go up and down stairs, and to run and jump. However, their gait velocity, balance, and motor coordination may be reduced compared to a paired child with normal development. Difference between children of GMFCS levels I and II included limitations in walking outdoors and in the community, on uneven surfaces, and in crowded places. Children of level II may hold onto a rail to climb stairs, may require wheeled mobility when traveling long distances, and their ability to perform gross motor skills such as running and jumping are minimal compared to children in level I [5]. Studies showing disagreement between levels I and II have been reported, suggesting difficulty in classifying if a child has functional limitation or can perform gross motor skills [5, 7].

During normal gait strike, the hemipelvis of the stance limb is aligned with the contralateral hemipelvis. At the beginning of loading response and mid-stance, the contralateral hemipelvis drops in the frontal plane, and the stance hemipelvis is higher around 4° to 7° [22]. The effect of pelvic drop is to adjust the length of the support lower limb, helped by some degree of knee flexion, avoiding excessive lower vertical displacement of the center of mass [23]. The hip abduction/adduction displacement at this moment is dependent on the pelvis movement over the femur [24]. While hemipelvis elevation favors adduction of the stance limb, hemipelvis drop favors, on the contralateral side, hip abduction movement [24]. Besides saving body energy by preventing greater inferior displacement of the center of mass, hip adduction of the stance limb, also shifts the body center of mass towards the center of rotation of the hip being loaded. The result would be a decrease in the external adductor moment of force, favoring the mechanical advantage of the hip abductor muscles during stance [25]. The summation of these actions prevents excessive pelvic drop and the Trendelenburg gait [22]. As well, when the support limb begins terminal stance and preswing phases, the opposite will occur. The pelvis will begin to drop, favoring hip abduction which is important to assist the release of the foot from the ground, permitting the body to swing forward [22, 24]. Therefore, pelvic obliquity and hip adduction are two mechanisms that work synchronously to maintain stability and forward movement of the body.

In diplegic children with cerebral palsy, reduced pelvic obliquity could be a result of abductor muscles weakness bilaterally and adductor muscles spasticity [26]. Another explanation could be that, by reducing pelvic elevation on the supported limb in the stance phase of the cycle, children with cerebral palsy are probably trying to decrease leg length to facilitate the next initial contact of the same foot [26]. However, this strategy would increase inferior displacement of the center of mass cycle, resulting in a less efficient gait [25, 27].

At the hip joint, reduced hip adduction in the stance phase increases the internal abductor moment of the support limb [28]. Since children with cerebral palsy normally show weakness of the hip abductor muscles, the gait pattern would be unstable, forcing them to increase base of support or to spend less time in the unipodal phase [14, 22]. The decreased hip adduction could also be a response to alterations in pelvic obliquity, once the loaded hemipelvis is lower than normal, challenging the hip mechanics to generate hip adduction.

The combination of reduced pelvic obliquity and hip adduction during the stance phase of the gait cycle, reinforce the evidence that children with cerebral palsy GMFCS level II are more unstable during gait compared to children in level I. In threatening situations, such as walking on uneven or inclined surfaces, the greater instability of children in GMFCS level II could justify their need for assistive devices. It also justifies their use a rail to walk up and down stairs, since reduced pelvic obliquity would decrease hip abduction that could also affect hip flexion. Although the kinematic analysis was not a tool used to assist the development of GMFCS classification system, the findings of the present study support the differences between levels I and II of the GMFCS, recently revised by the authors [29].

The discriminant model revealed that pelvic obliquity has a higher impact in discriminating children GMFCS level I from those of level II. Clinically, this result shows that pelvic obliquity in the frontal plane explains better the mechanical difference between children in level I and II. The importance of pelvic obliquity during gait was introduced in 1953 by Saunders and coworkers [30] as one of the most important gait marker that minimizes the vertical displacement of the center of mass. In 2001, Della Croce [25] and coworkers confirmed that pelvic obliquity and single support knee flexion are the second most important gait determinants in reducing center of mass dislocation and consequently improving gait efficiency [25].

The present study demonstrated that gait velocity was similar between CP children in level I and II. Bagley et al. [8], in a multicentre study conducted with 562 children with cerebral palsy, classified in levels I to III of the GMFCS, determined that gait velocity is a discriminant factor between levels I and II. Similar results were reported by Oeffinger et al. [1]. In addition, Damiano and Abel [31] reported that the temporal and spatial parameters were important indicators of the severity level of the cerebral palsy children. The classification in levels I and II, proposed by Palisano et al. [5], was based primarily in the limitation of the child to execute movements that involved velocity and stability, such as walking, jumping, and going up and down stairs. Therefore, we would expect a significant difference between levels I and II in the gait spatial and temporal markers. It is likely that the different instruments used to capture temporal and spatial gait markers and the fact that our sample was composed only of diplegic cerebral palsy children probably justify the different results found in the present study.

Gait alterations in children with cerebral palsy may also occur in other planes of motion. Rodda and Graham [32] proposed a classification system for diplegic children based on the kinematic and kinetic analyses focused in the sagittal plane. However, the authors agreed that important alterations may also occur in the frontal and transverse planes. For example, excessive internal pelvic rotation during initial contact contributes to a higher range of hip abduction motion observed during gait strike. On the other hand, increased pelvic obliquity may be secondary to a decrease in the hip and knee sagittal motions. The lack of significant findings in the sagittal plane could be related to the nature of the movement. As in the sagittal plane, the range of motion is greater compared to the other planes, the variability is normally smaller when compared to movements with smaller range of motions [33, 34]. One option would be to increase the sample size, in an attempt to identify the subtle variances that could occur in the sagittal plane. Nevertheless, in the present study, even with a relatively small sample size the results could discriminate the groups, showing that, in fact, a difference between levels I and II, although subtle, could be detected with the multivariate analysis technique applied.

The present study offers new information on angular displacement in all three planes of gait stance of children with cerebral palsy classified as GMFCS levels I and II. Clinical observation of reduced pelvic obliquity and hip adduction during gait is a difficult task. The literature on observational gait analysis has shown that training and experience are important for a more consistent observation of the pelvic and hip movements during gait [35]. Therefore, the kinematic idiosyncrasy of each GMFCS level could be added as one important clinical parameter to help the classification process of mild-to-moderate children with diplegia. In addition, prior knowledge of the biomechanical differences between GMFCS levels I and II may guide further physical therapy strategies, focused on regaining pelvic obliquity and hip adduction range of motion of the support limb. Such strategies may promote gait stability of cerebral palsy children level II, with less energy expenditure and free from assistive devices. 

## Figures and Tables

**Table 1:** Mean (SD) or frequency (%) and mean difference (95% CI) of the anthropometric characteristics between the groups of children with diplegia levels I and II of the GMFCS.

Characteristic	Groups		Difference between groups
	Level I	Level II	Level I minus Level II
	*N* = 11	*N* = 11	
Age (year) mean (SD)	9.1	9.8	−0.7
	(2.3)	(2.1)	(−2.7 to 1.3)
Height (m), mean (SD)	1.3	1.3	0
	(0.1)	(0.1)	(−0.1 to 0.1)
BMI (Kg/m^2^)	16.7	17.2	−0.3
	(2.0)	(3.8)	(−3.2 to 2.2)
Sex (%)			
Females	3	4	—
Males	8	7	—

**Table 2:** Mean (SD) and mean difference (95% CI) of the temporal and spatial variables between the groups of children with diplegia levels I and II of the GMFCS.

Outcomes	Groups		Difference between groups
	Level I	Level II	Level I minus Level II
	*N* = 11	*N* = 11	
Velocity (m/s), mean (SD)	0.7	0.6	0.1
	(0.2)	(0.2)	(−0.1 to 0.3)
Stride length (m), mean (SD)	0.8	0.7	0.1
	(0.1)	(0.2)	(−0.04 to 0.2)
Time (s), mean (SD)			
Cycle	1.1	1.1	0
	(0.2)	(0.2)	(−0.2 to 0.2)
Stance	0.6	0.6	0
	(0.1)	(0.1)	(−0.1 to 0.1)
Swing	0.5	0.5	0
	(0.1)	(0.2)	(−0.14 to 0.14)

**Table 3:** Mean (SD) and *P*-values of the principal components from the pelvis and hip joint in the frontal plane during the stance phase of the gait cycle between the groups of children with diplegia levels I and II of the GMFCS.

Principal components	Groups GMFCS		*P*-value
	Level I	Level II
	(*N* = 11)	(*N* = 11)
Frontal plane			
Pelvis			
PC1	−33.9	5.1	^ ∗^0.02
	(40.9)	(31.6)	
PC2	−11,7	1.6	0.07
	(11.2)	(19.8)	
Hip			
PC1	−64.6	−8.5	^ ∗^0.03
	(31.9)	(71.1)	
PC2	−28.3	−14.1	0.08
	(20.2)	(15.9)	

*Significant at *P* < 0.05.
